# Ultrasensitive Kilo-Pixel Imaging Array of Photon Noise-Limited Kinetic Inductance Detectors Over an Octave of Bandwidth for THz Astronomy

**DOI:** 10.1007/s10909-018-1962-8

**Published:** 2018-05-29

**Authors:** J. Bueno, V. Murugesan, K. Karatsu, D. J. Thoen, J. J. A Baselmans

**Affiliations:** 1 SRON Netherlands Institute for Space Research, Utrecht, The Netherlands; 2 Terahertz Sensing Group, Delft University of Technology, Delft, The Netherlands

**Keywords:** Kinetic inductance detectors, Kilo-pixel array, THz astronomy

## Abstract

We present the development of a background-limited kilo-pixel imaging array of ultrawide bandwidth kinetic inductance detectors (KIDs) suitable for space-based THz astronomy applications. The array consists of 989 KIDs, in which the radiation is coupled to each KID via a leaky lens antenna, covering the frequency range between 1.4 and 2.8 THz. The single pixel performance is fully characterised using a representative small array in terms of sensitivity, optical efficiency, beam pattern and frequency response, matching very well its expected performance. The kilo-pixel array is characterised electrically, finding a yield larger than 90% and an averaged noise-equivalent power lower than 3 $\times$ 10$^{-19}$ W/Hz$^{1/2}$. The interaction between the kilo-pixel array and cosmic rays is studied, with an expected dead time lower than 0.6% when operated in an L2 or a similar far-Earth orbit.

## Introduction

The next generation of space-based imaging spectrometers for sub-millimetre (sub-mm) wave astronomy requires broad band radiation coupling between 1 and 10 THz [1, 2]. These spectrometers will allow measurements of a large number of spectroscopic bands over a wide area of the sky in a very limited time. In order to do so, they will require a large number of pixels to cover the telescope field of view or to sample a given frequency band with a high resolution. Kinetic inductance detectors (KIDs) are superconducting pair-breaking resonators [3] that are a very attractive choice for these applications since thousands of detectors can be read out with a single coaxial line [3, 4], enabling simple and cost-effective systems. Since these spectrometers can only be used from space at these high frequencies, the requirements on the detector sensitivity [5] are extremely demanding, typically with an noise-equivalent power (NEP) of $\sim$ 3 $\times$ 10$^{-19}$ W/Hz$^{1/2}$ for a non-dispersive spectrometer. Such sensitivities have been achieved with antenna-coupled aluminium (Al) KIDs over a broad band [6] around 1.5 THz with poor beam quality and over a narrow band around 850 GHz [4, 7]. In this paper, we extend KID technology to higher frequencies and large bandwidths using a leaky lens antenna-coupled device. This device allows high coupling efficiency over an octave of bandwidth at frequencies higher than 1 THz.

## Design and Fabrication

We have designed, fabricated and measured a small chip of leak-lens antenna-coupled KIDs operating in the 1.4–2.8-THz band [8]. The KID design combines the hybrid NbTiN/Al technology to obtain good noise performance [9] and the all-Al antenna concept [6] to provide a very high sensitivity. A long and detailed discussion about the requirements of the detector system, its fabrication and full characterisation (sensitivity, optical efficiency, beam pattern and frequency response) is presented in our previous work [8]. In summary, the device has a beam pattern and frequency response close to the simulated parameters and has a limiting sensitivity given by a NEP$_\mathrm{opt}$ $=$ 2.5 $\times$ 10$^{-19}$ W/Hz$^{1/2}$.

**Fig. 1:**
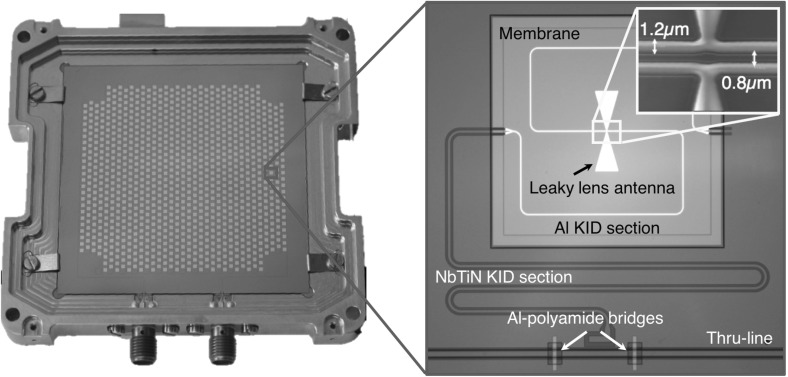
Image of the kilo-pixel leaky lens antenna-coupled KID array. Left: photograph of the array mounted in its holder. Right: back- and front-illuminated optical image of a single pixel of the leaky lens antenna-coupled KID. The light goes through the membrane where both the antenna and the Al section of the KID are fabricated. The centre of the antenna is shown as an inset with an SEM image (Colour figure online)

In this paper we focus on the scalability of the single pixel device into a kilo-pixel array. All the fabrication details are discussed in our previous work [8], and the same process flow is followed in the fabrication of the device presented in this paper. An image of the fabricated kilo-pixel leaky lens antenna-coupled KID array is shown in Fig. 1. The detector array consists of 989 pixel KIDs hexagonally packed, with a pixel spacing of 1.6 mm covering an area of 48 $\times$ 48 mm on a 55 $\times$ 55 mm chip.

The THz radiation is coupled to the leaky slot in the Al ground plane, which launches the radiation into the two very narrow Al CPW lines. The length of the Al lines ($\sim$1.25 mm) is such that all THz radiation is absorbed over the whole octave of bandwidth before the lines become wide. The length of the Al has been chosen to absorb more than 10 dB of power for the highest radiation frequency (2.8 THz) before reaching the NbTiN evaluating the attenuation constant of the line using CST. The Al line absorbs even more at the lowest frequency (1.4 THz). The narrow linewidth (0.8 $\upmu$m strip with a 1.2 $\upmu$m gap) is needed to limit radiation loss. The narrow Al line broadens at either end and connects to a wide NbTiN CPW (strip of 12 $\upmu$m with a gap of 8 $\upmu$m). The NbTiN central conductor is shorted to the NbTiN ground at the far end of the resonator. At the other end, the NbTiN remains wide is deposited on the bare Si substrate for most of its length. The main challenge of the fabrication is to resolve the narrow aluminium line (1.2–0.8–1.2 $\upmu$m) close to the antenna with a high yield across the whole wafer.

## Electrical Characterisation

A 3D assembly of the detector chip, spacer wafer and lens array is needed to couple radiation efficiently to the device [8]. It is crucial to reach a vacuum gap between the antenna and the spacer wafer of less than 6 $\upmu$m, which is very challenging for a 55 $\times$ 55 mm chip (like the one presented in this paper). A smaller prototype with 19 pixels has been characterised under radiation-loaded conditions, showing very good sensitivity, optical efficiency, beam pattern quality and broad frequency response [8]. In this work we limit ourselves to a dark measurement of the kilo-pixel array, which is possible using a measurement of the detector chip only, without spacer wafer and lens array. We take advantage of the fact that for NbTiN-Al hybrid KIDs it has been proven that the electrical NEP is a very good approximation for the optical NEP [4, 10].

**Fig. 2:**
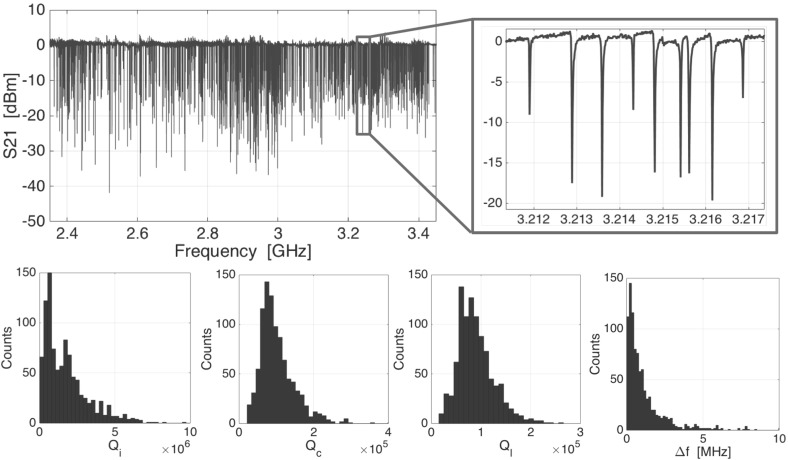
Top: frequency response of the kilo-pixel array of leaky lens antenna-coupled KIDs taken at a temperature of 120 mK. The zoom shows a few resonators, the relative bandwidth of the resonators and the scatter in frequency of the resonators. Bottom: histograms of the internal, coupling and loaded quality factors of the array and the frequency separation between resonators (Colour figure online)

**Fig. 3:**
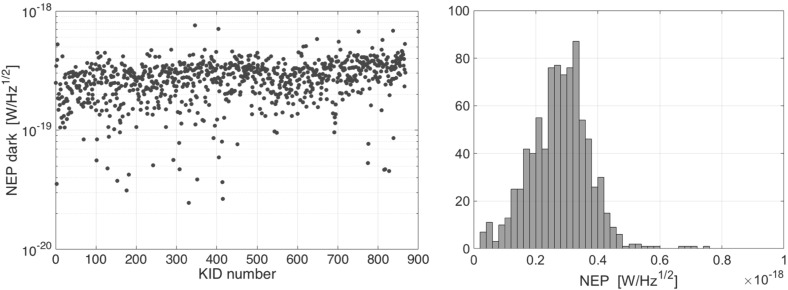
Electrical characterisation of the kilo-pixel array of leaky lens antenna-coupled KIDs. Left: dark NEP for all MKIDs of the array, obtained by measuring the temperature response of the chip and the noise spectra at 120 mK. Right: histogram of the data plotted. The averaged electrical (dark) NEP is 3 $\times$ 10$^{-19}$ W/Hz$^{1/2}$ (Colour figure online)

To characterise the performance of the kilo-pixel detector array we mount it in a closed sample holder in a ‘box-in-a-box’ configuration on the cold stage of an adiabatic demagnetisation refrigerator (ADR) [11], where the temperature of the chip is stabilised at 120 mK. We use a commercial vector network analyser to measure the forward scattering parameter S21 of the system as a function of frequency. The results are shown in Fig. 2. Multiple dips in the transmission appear, each of them corresponding to an individual KID. All the resonances are placed in a frequency span of 1.2 GHz centred at 2.9 GHz. We find 915 resonators out of 989, which corresponds with a fabrication yield of 93%. The fit to all the resonance features shows that we obtain an average loaded *Q* factor $Q=90\times 10^{3}$, an average coupling *Q* factor $Q_{c} = 100 \times 10 ^{3}$ and an average internal *Q* factor $Q_{i} = 1.8 \times 10 ^{6}$.

We measure the electrical (dark) NEP of the detectors using the method described in Baselmans et al. [12], reading out 860 pixels of the array simultaneously using frequency division multiplexing [13]. The response of the KIDs to a change in chip temperature is measured. The amount of quasiparticles in the aluminium $N_\mathrm{qp}$ can be calculated from the chip temperature, the volume of the aluminium section of the resonator and the energy gap. The dark NEP using the phase read-out is shown in Fig. 3. We find an average value of the electrical NEP is given by NEP$_\mathrm{dark} = 2.8 \pm 1\times 10 ^{-19}$ W/Hz$^{1/2}$ for the phase read-out. The scatter in the NEP between the pixels is a result of fabrication inaccuracies resulting in a spread of the aluminium properties over the wafer.

The electrical NEP values are in excellent agreement with the optical NEP measured at 1.55 THz presented in our previous characterisation of a single pixel [8], which confirms that the dark NEP is a good measurement of the detector sensitivity [4, 8, 10]. We can expect that a full-size lens array coupled to the presented chip would result in a imaging array with a limiting sensitivity given in Fig. 3.

**Fig. 4:**
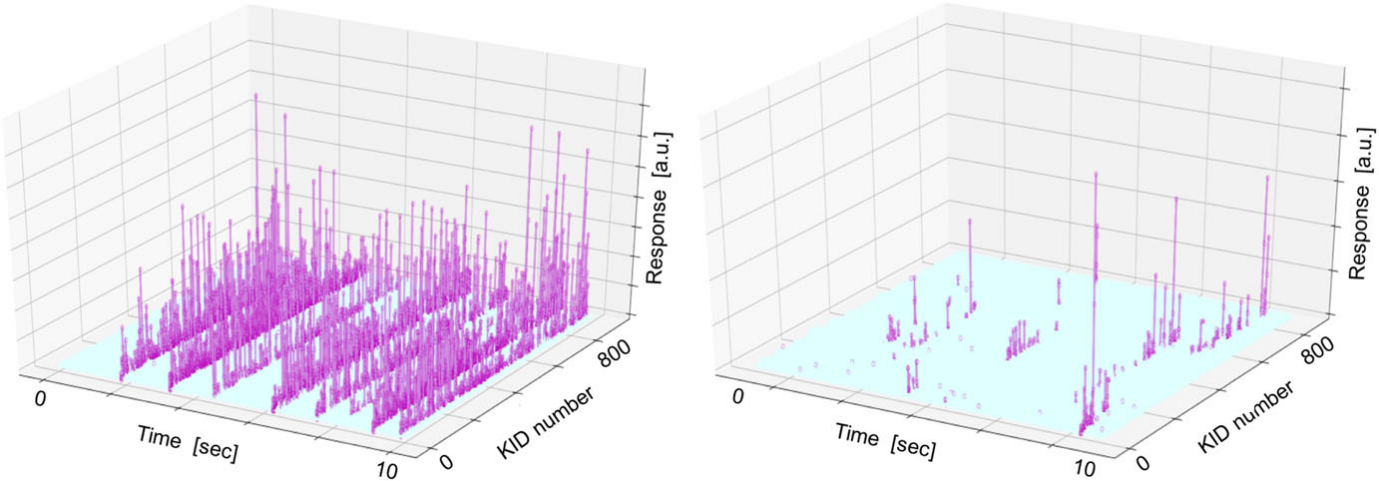
Susceptibility of kilo-pixel arrays of antenna-coupled KIDs to cosmic rays. Left: time trace of 10 s of a kilo-pixel KIDs array on a solid substrate. All KIDs simultaneously are affected by a cosmic ray hit. Right: time trace of 10 s of a kilo-pixel KIDs array with membranes. It is clear that less KIDs are affected simultaneously by a cosmic ray hit (Colour figure online)

## Interaction Between the Detector Array and Cosmic Rays

Space observatories operating outside low-Earth orbit are subject to interactions with cosmic rays, which are so energetic that it is impossible to effectively shield the detector arrays. Thus, cosmic rays will inevitably interact with the detector chip, thereby depositing a fraction of their energy by ionisation and atomic excitation. The typical result of a cosmic ray interaction is a glitch that results in difficult data retrieval and loss of integration efficiency [14]. We evaluate the effect of cosmic ray interactions in the detector chip by measuring the effects of secondary cosmic rays, which are a result from the interaction of primary cosmic rays with the Earths atmosphere. The primary cosmic rays mainly consist of protons, whereas the main component of secondary cosmic rays is muons. Muons easily penetrate into shields of the setup and create glitches during experiments.

We have to remove the resulting glitches in all our experiments to obtain the results presented in the previous section. We use an iterative de-glitching scheme to do so, which consists of a few steps: (i) we calculate 2nd derivative of time-ordered data to enhance the glitches as well as removing slow drifts in the data; (ii) we calculate the rms of the 2nd derivative data and then remove points where the rms value is larger than $6~\sigma$. With this step, we identify large glitches that correspond to cosmic rays with large energy deposition on the chip; (iii) we calculate the rms of data from the previous step (i.e. 2nd derivative data from which large glitches are already removed) and identify points where the rms value is larger than $5~\sigma$. We identify small glitches with this step; (iv) the identified points at (ii) and (iii) are removed from the original data set to create de-glitched data.

In order to study the cosmic ray effects in detail, we take 30 min of data with the read-out system in its fast, low-resolution setting with a sampling rate of 1.27 kHz and an integration time of 787 $\upmu$s while operating the chip in dark conditions. There is not dead time in the measurement because the data were taken with 2 GHz speed with no interruption and cosmic rays are tagged afterwards. A typical 10 s time trace for two different kilo-pixel arrays of antenna-coupled KIDs is shown in Fig. 4, one made on a solid substrate and the other one with membranes (for the leaky lens design). The timeline of the KIDs is clearly affected by the glitches. In the solid substrate array all the glitches are seen by most of the KIDs, whereas in the array with membranes the glitches do not spread very much and only a few KIDs are affected per glitch.

We obtain a count rate for muons of 0.8 events/s/(5.5 cm)$^{2}\,\times$ 60 s/min $\sim$ 1.6 event/cm$^{2}$/min for the kilo-pixel KIDs array on a solid substrate, which is consistent with the standard value of 1 event/cm$^{2}$/min. We estimate the effect of cosmic ray interactions when operating the array in L2 based on these measurements by scaling the hit rate on the chips to the measured event rate from Planck of 5 events/s/cm$^{2}$. This simple scaling results in an estimated loss in integration time about 0.6% for the leaky lens design that is $\sim$30 times smaller than the solid substrate array. It is possible to harden KID arrays against cosmic ray events by adding a layer of a superconducting material with a critical temperature below the one of the aluminium of the KIDs [15]. The non-thermal (high-energy) phonons created by the initial interaction and subsequent phonon downconversion are converted to phonons with an energy *E* < 2$\varDelta _\mathrm{Al}$ through electron–phonon interactions in the low-temperature superconducting layer. This technique has been used successfully in a similar size array of KIDs resulting in an estimated loss in integration time of 4% [4]. Although the high-energy phonon-absorbing layer of superconducting material improves the loss in integration time, the leaky lens-coupled KID array is still 7 times better. Further experiments to determine the mechanism that prevents the spreading of the glitch effect have been carried out [16].

## Conclusions

A 989 pixels of leaky lens antenna-coupled KID imaging system providing an octave of bandwidth between 1.4 and 2.8 THz have been fabricated with a fabrication yield of 93%. The system is read out using a single set of read-out electronics and one pair of coaxial cables. This kilo-pixel array has been characterised electrically sweeping the temperature of the array, with an average sensitivity of NEP$_\mathrm{dark} = 2.8 \times 10^{-19}$ W/Hz$^{1/2}$. The electrical and optical (measured in a separate smaller array) sensitivities are identical. Additionally, the detector array is not very sensitive to cosmic ray interaction with an expected loss of integration time of less than 0.6% when operated in L2 orbit. This device and assembly can be scaled and used for the higher frequency bands (up to 10 THz) of SPICA-SAFARI [1] after implementing some modifications: (i) the CPW section of the KID close to the antenna needs to be made with electron beam lithography in order to make $\sim$300-nm lines; (ii) the gap between the lens and the antenna needs to be reduced down to 1 $\upmu$m, which can be done using a spinnable and patternable bonding adhesive; (iii) the alignment between the lens and the antenna needs to be improved down to a few $\upmu$m, which can be done using micromachined Si springs [17]. In summary, this array fulfils many generic requirements for future THz and sub-mm wave space-based observatories.
